# Prone positioning in non-intubated patients with COVID-19 associated acute respiratory failure, the PRO-CARF trial: A structured summary of a study protocol for a randomised controlled trial

**DOI:** 10.1186/s13063-020-04882-2

**Published:** 2020-11-23

**Authors:** Miguel Á. Ibarra-Estrada, Miguel Marín-Rosales, Roxana García-Salcido, Sara A. Aguirre-Díaz, Alexandra Vargas-Obieta, Quetzalcóatl Chávez-Peña, José A. López-Pulgarín, Julio C. Mijangos-Méndez, Guadalupe Aguirre-Avalos

**Affiliations:** 1 Intensive Care Unit, COVID-19 Unit, Hospital Civil Fray Antonio Alcalde, Guadalajara, Jalisco Mexico; 2 Internal Medidine, COVID-19 Unit, Hospital General de Occidente, Zapopan, Jalisco Mexico; 3 COVID-19 Unit, Hospital Civil Fray Antonio Alcalde, Guadalajara, Jalisco Mexico; 4 Infectious Diseases Department, Hospital Civil Fray Antonio Alcalde, Guadalajara, Jalisco Mexico; 5 Centro Universitario de Ciencias de la Salud, Universidad de Guadalajara, Guadalajara, Jalisco Mexico; 6 Intensive Care Unit, COVID-19 Unit, Hospital Civil “Fray Antonio Alcalde”, Guadalajara, Jalisco Mexico

**Keywords:** COVID-19, randomised controlled trial, protocol, awake prone-positioning, acute respiratory failure, high-flow nasal cannula, endotracheal intubation

## Abstract

**Objectives:**

To assess the effect of prone positioning therapy on intubation rate in awake patients with COVID-19 and acute respiratory failure.

**Trial design:**

This is a two-center parallel group, superiority, randomized (1:1 allocation ratio) controlled trial.

**Participants:**

All patients admitted to the Hospital Civil de Guadalajara and Hospital General de Occidente in Mexico for COVID-19 associated acute respiratory failure and in need of supplementary oxygen through high-flow nasal cannula are screened for eligibility.

Inclusion criteria: all adult patients admitted to the COVID-19 unit who test positive for COVID-19 by PCR-test and in need for oxygen are eligible for inclusion. Randomization starts upon identification of requirement of a fraction of inspired oxygen ≥30% for an oxygen capillary saturation of ≥90%

Exclusion criteria: less than 18 years-old, pregnancy, patients with immediate need of invasive mechanical ventilation (altered mental status, fatigue), vasopressor requirement to maintain median arterial pressure >65 mmHg, contraindications for prone positioning therapy (recent abdominal or thoracic surgery or trauma, facial, pelvic or spine fracture, untreated pneumothorax, do-not-resuscitate or do-not-intubate order, refusal or inability of the patient to enroll in the study.

**Intervention and comparator:**

Patients of the intervention group will be asked to remain in a prone position throughout the day as long as possible, with breaks according to tolerance. Pillows will be offered for maximizing comfort at chest, pelvis and knees. Monitoring of vital signs will not be suspended. Inspired fraction of oxygen will be titrated to maintain a capillary saturation of 92%-95%. For patients in the control group, prone positioning will be allowed as a rescue therapy. Staff intensivists will monitor the patient’s status in both groups on a 24/7 basis. All other treatment will be unchanged and left to the attending physicians.

**Main outcomes:**

Endotracheal intubation rate for mechanical ventilation at 28 days.

**Randomisation:**

Patients will be randomly allocated to either prone positioning or control group at 1:1 ratio. Such randomization will be computer generated and stratified by center with permuted blocks and length of 4.

**Blinding (masking):**

Due to logistical reasons, only principal investigators and the data analyst will be blinded to group assignment.

**Numbers to be randomised (sample size):**

With an intubation rate of 60% according to recent reports from some American centers, and assuming a decrease to 40% to be clinically relevant, we calculated a total of 96 patients per group, for a beta error of 0.2, and alpha of 0.5. Therefore, we plan to recruit 200 patients, accounting for minimal losses to follow up, with 100 non-intubated patients in the prone position group and a 100 in the control group.

**Trial Status:**

The local registration number is 048-20, with the protocol version number 2.0. The date of approval is 3rd May 2020. Recruitment started on 3^rd^ May and is expected to end in December 2020.

**Trial registration:**

The protocol was retrospectively registered under the title: “Prone Positioning in Non-intubated Patients With COVID-19 Associated Acute Respiratory Failure. The PRO-CARF trial” in ClinicalTrials.gov with the registration number: NCT04477655. Registered on 20 July 2020.

**Full protocol:**

The full protocol is attached as an additional file, accessible from the Trials website (Additional file 1). In the interest in expediting dissemination of this material, the familiar formatting has been eliminated; this Letter serves as a summary of the key elements of the full protocol.

The study protocol has been reported in accordance with the Standard Protocol Items: Recommendations for Clinical Interventional Trials (SPIRIT) guidelines (Additional file 2).

**Supplementary Information:**

The online version contains supplementary material available at 10.1186/s13063-020-04882-2.

## Supplementary Information

**Additional file 1**

**Additional file 2**

## Data Availability

The final dataset will be available to the research-team, to the local authorities and upon reasonable request to others after agreement of the local IRB. All data are anonymised and stored safely for five years according to local law.

