# Supplementary material for: Prone positioning in non-intubated patients with COVID-19 associated acute respiratory failure, the PRO-CARF trial: A structured summary of a study protocol for a randomised controlled trial
Source: Trials. 2020 Nov 23;21:940. doi: 10.1186/s13063-020-04882-2 (PMC7680981; doi:10.1186/s13063-020-04882-2)
Supplement: Supplementary file 1 — Additional file 1 [file 13063_2020_4882_MOESM1_ESM.pdf]

Prone Positioning in Non-intubated Patients With COVID-19 Associated Acute Respiratory Failure (PRO-CARF)

Protocol Version. 2.0 (May 3rd, 2020)

-Miguel Á. Ibarra-Estrada. Intensive Care Unit, COVID-19 Unit, Hospital Civil Fray Antonio Alcalde. Guadalajara, Jalisco.
-Miguel Marín-Rosales. Internal Medicine, COVID-19 Unit, Hospital General de Occidente. Zapopan, Jalisco.
-Roxana García-Salcido. COVID-19 Unit. Hospital Civil Fray Antonio Alcalde. Guadalajara, Jalisco.
-Sara A. Aguirre-Díaz. Infectious Diseases Department, COVID-19 Unit, Hospital Civil Fray Antonio Alcalde. Guadalajara, Jalisco.
-Alexandra Vargas-Obieta. Intensive Care Unit, COVID-19 Unit, Hospital Civil Fray Antonio Alcalde. Guadalajara, Jalisco.
-Quetzalcóatl Chávez-Peña. Intensive Care Unit, COVID-19 Unit, Hospital Civil Fray Antonio Alcalde. Guadalajara, Jalisco. Centro Universitario de Ciencias de la Salud, Universidad de Guadalajara, Jalisco.
-José A. López-Pulgarín. Intensive Care Unit, COVID-19 Unit, Hospital Civil “Fray Antonio Alcalde”. Guadalajara, Jalisco. Centro Universitario de Ciencias de la Salud, Universidad de Guadalajara, Jalisco.
-Julio C. Mijangos-Mendez. Intensive Care Unit, COVID-19 Unit, Hospital Civil Fray Antonio Alcalde. Guadalajara, Jalisco.
-Guadalupe Aguirre-Avalos. Intensive Care Unit, COVID-19 Unit, Hospital Civil “Fray Antonio Alcalde”. Guadalajara, Jalisco. Centro Universitario de Ciencias de la Salud, Universidad de Guadalajara, Jalisco.

Background:

On December 2019, many cases of unknown origin pneumonia appeared in Wuhan, Hubei, China, resembling viral pneumonia. Deep sequencing analysis demonstrated the presence of a new beta-coronavirus, termed SARS-CoV-2 (1).

Coronavirus-19 disease (COVID-19) is characterized by a rapid progression to respiratory failure after symptom onset. Most patients have criteria for acute respiratory distress syndrome (ARDS), consisting of acute radiographic infiltrates, hypoxemia, and lung edema of non-cardiac or fluid overload origin (2). However, many patients present with a mismatch between severity of hypoxemia and preserved lung mechanics. Therefore, ventilation-perfusion mismatch could predominate, which implies less alveolar collapse than expected, and response to regular therapies for ARDS may not be similar (3).

Despite general mortality of COVID-19 is less than 5%, it can reach up to 62% in critically-ill patients and even higher in patients with mechanical ventilation (4).

A still underused therapy but with proven benefit in patients with ARDS, is the prone positioning therapy, consisting of prone positioning of the patients everyday for prolonged periods, at least of 16 h, however it is only proven in mechanically ventilated patients (5).

There are scarce data regarding prone positioning in awake patients before COVI-19 pandemic. A retrospective study of 15 patients, showed this therapy combined with non-invasive mechanical ventilation significantly improved oxygenation, however this improvement was transient, as oxygenation returned to baseline at 6 h after prone sessions. There was not adverse events (6).

Another preceding study from china was published in 2019, this was a prospective observational trial which included 20 patients with moderate to severe ARDS. Authors found that prone positioning combined with oxygen through high-flow nasal cannula or non-invasive mechanical ventilation, improved significantly the pO2/FIO2 ratio, and half of the patients avoided intubation. However, according to the design of the study and low sample size, this can only be taken as hypothesis-generating data (7).

Regarding to prone positioning in COVID-19 patients, there are two observational reports, one of them with 24 (8) and the other with 15 patients (9), and only found a non-sustained improvement in oxygenation, and both had a short time to follow-up. The largest trial so far, is a feasibility prospective cohort (10),

which included 56 patients, and also showed an improvement in pO2/FiO2 ratio, with an increase of 50% after a prone session of 3 hours, the pO2/FiO2 ratio returned to baseline levels in half of the patients; moreover, intubation rate was not different between patients considered as responders (increase in pO2/FiO2 ratio) and non-responders. Therefore the question if this therapy can lower the requirement of mechanical ventilation is still open.

Although prone positioning is a relatively safe therapy, it is not a standard of care and is only recommended for intubated patients with moderate to severe ARDS according to guidelines (11). Therefore, we propose this randomized controlled trial, as the potential benefit is high, and it could help to optimize resource utilization in patients with this COVID-19, as we foresee the maximal peak of the pandemic at our region is still far from now.

Objectives

Primary Objective

The primary outcome will be intubation rate for mechanical ventilation at 28 days.

Secondary objectives

The secondary outcomes will be:

1. Total hours of prone position at day, < at 28 days.
2. All laboratory variables recorded from admission to discharge.
3. Lung ultrasound (LUS) score at admission to COVID- unit
4. Total number of prone sessions at day, at 28 days.
5. Hours of the longest prone session each day, at 28 days.
6. Change in oxygenation 1-hour after first prone session.
7. Change in the ROX-index 1-hour after first prone session.
8. Total days of prone positioning therapy, at 28 days.
9. Adverse effects of prone positioning therapy (ulceration, back pain, intravenous lines dislodgement), at 28 days.
10. Mechanical ventilation days, at 28 days.
11. Intensive care unit length of stay, at 28 days.
12. Hospital length of stay, at 28 days.

13. Hospital mortality, 28 days.

Methods

This is a multi-center, parallel, superiority, open label, randomized controlled trial, registered with ClinicalTrials.gov (NCT04477655). It will be approved by the Ethic Committees of both participant hospitals. And informed consent will be obtained from all patients.

Inclusion criteria

-Regardless of diagnosis of acute respiratory distress syndrome, all adult patients (<18 y) patients with confirmed COVID-19 by PCR and respiratory distress will be included.

-Requirement of a fraction of inspired oxygen (FiO2) ≥30% through high-flow nasal cannula (HFNC) to maintain a capillary saturation of ≥90%.

Exclusion criteria

-Less than 18 years-old

-Pregnancy

-Patients with immediate need of invasive mechanical ventilation (altered mental status, fatigue)

-Vasopressor requirement to maintain median arterial pressure >65 mmHg

-Contraindications for prone positioning therapy (recent abdominal or thoracic surgery or trauma, facial, pelvic or spine fracture, untreated pneumothorax.

-Do-not-resuscitate or do-not-intubate order

-Refusal or disability of the patient to enroll in the study

Recruitment

All patients admitted with COVID-19 to intermediate and/or intensive care unit at participating hospitals (any oxygen requirement) will be screened at two academic hospitals (Hospital Civil de Guadalajara and Hospital General de Occidente, Guadalajara, Jalisco, México), and patients with requirement of HFNC with FiO2 ≥30% will be approach to participate by on-site critical care physicians. In case of

absence of exclusion criteria, written informed consent will be obtained before randomization.

Procedures (arms):

Patients of the control group will be treated with oxygen therapy through high flow nasal cannula (HFNC). Continuous monitoring of vital signs. Inspired fraction of oxygen will be titrated to maintain a capillary saturation of 92%-95%. Prone positioning will be allowed as a rescue therapy.

Patients of the experimental group will be also treated with oxygen therapy through high flow nasal cannula (HFNC). Patients will be asked to remain in prone position throughout the day as long as possible, with breaks according to tolerance. Pillows will be offered for maximizing comfort at chest, pelvis and knees. Monitoring of vital signs will not be suspended. Inspired fraction of oxygen will be titrated to maintain a capillary saturation of 92%-95%.

Staff intensivist will monitor adherence to protocol and patient's status of both groups on a 24/7 basis.

The prone positioning failure criteria will be:

-Worsening respiratory failure, defined as RR ≥40 /min and/or muscle fatigue

-Intolerance for any reason referred by the patient.

-Requirement of vasopressor to maintain MAP ≥65 mmHg

-Altered mental status

The decision for withhold prone positioning and proceed to endotracheal intubation will be left at the discretion of the attending intensivist. (see figure 1)

HFNC therapy will be initiated at 40 L/min and 37 ºC according to patient comfort and tolerance (Vapotherm, Precision flow, Exeter, New Hampshire), with FiO2 titrated to a capillary saturation ≥92%, and will be continuously delivered until stopping criteria are met. The primary criteria for stopping the prone positioning therapy will be a requirement of a FiO2 ≤40% to maintain a capillary saturation ≥90% AND a persistent ROX index of >4.87 for at least 2 hours after the last prone session.

Randomization and data monitoring

Patients will be randomly allocated to either prone positioning or control group at 1:1 ratio. Such randomization will be stratified by center with permuted blocks and length of 4; numbers will be generated by computer and patients will be sequentially numbered. Investigators assistants will recruit patients, will assign allocation groups, and will obtain informed consent from participants.

Blinding

Due to logistic reasons and the nature of the intervention, only investigators and data analysts will be blinded.

Management of data

A steering local committee will monitor the trial, with assessment of inputs to database for consistence and the presence of missing data. Study coordinators at both centers will verify accuracy of database inputs. On-site investigators at each center are responsible for adherence to the protocol, and filling paper and electronic case-report forms (see appendix). All investigators will be blinded regarding patient allocation and outcome measures until database unlock. All personal data will be coded and de-identified, and paper CRF will be stored for at least 5 years into locked cabinets at each center.

This trial is expected to initiate on May and completion is estimated in December 30th. As this study includes only hospitalized patients, lost to follow-up is not expected, however, all analysis will be performed on an intention-to-treat basis in the case of protocol violations.

Data monitoring committee will be composed of all investigators, and they will be responsible for data monitoring at the primary level, whereas the local IRB will monitor the overall conduct of the study.

Adverse effects

All potential adverse effects are expected to be non-serious (ulceration, back pain, intravenous lines dislodgment), however, all related adverse effects will be documented

for each patient, and all investigators will be notified monthly. In case of serious harm, the monitoring committee will discuss the termination of the study.

Statistical details

Sample size

With an intubation rate of 60% according to a recent report from some American centers , and assuming a decrease to 40% to be clinically relevant, we calculated at least a total of 96 patients per group, for a beta of 0.2, and alpha of 0.5. Therefore, we plan to recruit 200 patients, accounting for minimal losses to follow up. There are no plan for interim analysis.

Analysis

Categorical variables are presented as numbers and percentages, and comparison between groups will be performed with the chi-square test or the Fisher's exact test as appropriate. Continuous variables are given as means (±SD) or medians (inter-quartile ranges) and will be compared using the Student's t test or the Mann-Whitney test, according to Shapiro-Wilk test for distribution. For comparison between variables recorded at multiple time points, ANOVA for repeated measures will be used. The comparison between groups for cumulative probability of avoiding endotracheal intubation will be performed with Kaplan-Meier analysis and log-rank test. Analysis with ROC curve will be performed with prone positioning hours/day, taking endotracheal intubation as an endpoint. All tests will be performed at two-tails and a p value <0.05 will be considered as significant. All statistical analysis and graphics will be performed with MedCalc statistical software
(Ostend, Belgium).

Ethics approval

This study will initiate immediately after the approval from the institutional review boards at each hospital.

Protocol amendments

One of the investigators (GAA) will be responsible for documentation of the protocol amendments, and for process the approval from the IRB.

Conflict of interests

All authors hereby declare that they not have any conflict of interest

Access to data

Only investigators and IRB audit tema will have access to data.

Dissemination policy

Relevant clinical data obtained in this study will be published in peer-review journals of critical/respiratory care, without disclosing any individual patient data. All the investigators will be authors in those final publications. Patient level data, could be shared to abroad investigators on a reasonable request, after data sharing agreement is granted from the local IRB. Sharing of statistical code is not planed.

References:

1. C H, Y W, X L, L R, J Z, Y H, et al. Clinical Features of Patients Infected With 2019 Novel Coronavirus in Wuhan, China. Lancet (London, England). 2020;395(10223).

2. Force ADT, Ranieri VM, Rubenfeld GD, Thompson BT, Ferguson ND, Caldwell E, et al. Acute respiratory distress syndrome: the Berlin Definition. JAMA. 2012;307(23):2526-33.

3. L G, D C, S R. COVID-19 Pneumonia: ARDS or Not? Critical care (London, England). 2020;24(1).

4. S M, CD G, RA F. Care for Critically Ill Patients With COVID-19. JAMA. 2020.

5. Guerin C, Reignier J, Richard JC, Beuret P, Gacouin A, Boulain T, et al. Prone positioning in severe acute respiratory distress syndrome. N Engl J Med. 2013;368(23):2159-68.

6. V S, G G, L C, A Z, S I, A L, et al. Prone Positioning Improves Oxygenation in Spontaneously Breathing Nonintubated Patients With Hypoxemic Acute Respiratory Failure: A Retrospective Study. Journal of critical care. 2015;30(6).

7. L D, L W, W M, H H. Efficacy and Safety of Early Prone Positioning Combined With HFNC or NIV in Moderate to Severe ARDS: A Multi-Center Prospective Cohort Study. Critical care (London, England). 2020;24(1).

8. X E, Y T, AM D, F T, S M, E Ph, et al. Use of Prone Positioning in Nonintubated Patients With COVID-19 and Hypoxemic Acute Respiratory Failure. JAMA. 2020.

9. C S, M T, P S, A T, F C, G L, et al. Respiratory Parameters in Patients With COVID-19 After Using Noninvasive Ventilation in the Prone Position Outside the Intensive Care Unit. JAMA. 2020.

10. A C, G B, D W, M DP, A S, P F, et al. Feasibility and Physiological Effects of Prone Positioning in Non-Intubated Patients With Acute Respiratory Failure Due to COVID-19 (PRON-COVID): A Prospective Cohort Study. The Lancet Respiratory medicine. 2020.

11. W A, MH M, YM A, M L, MN G, E F, et al. Surviving Sepsis Campaign: Guidelines on the Management of Critically Ill Adults With Coronavirus Disease 2019 (COVID-19). Intensive care medicine. 2020.

12. RAA, ADK, TMC, et al. Outcomes from intensive care in patients with COVID-19: a systematic review and meta-analysis of observational studies. Anaesthesia 2020 Oct;75(10):1340-1349.

Recruitment process

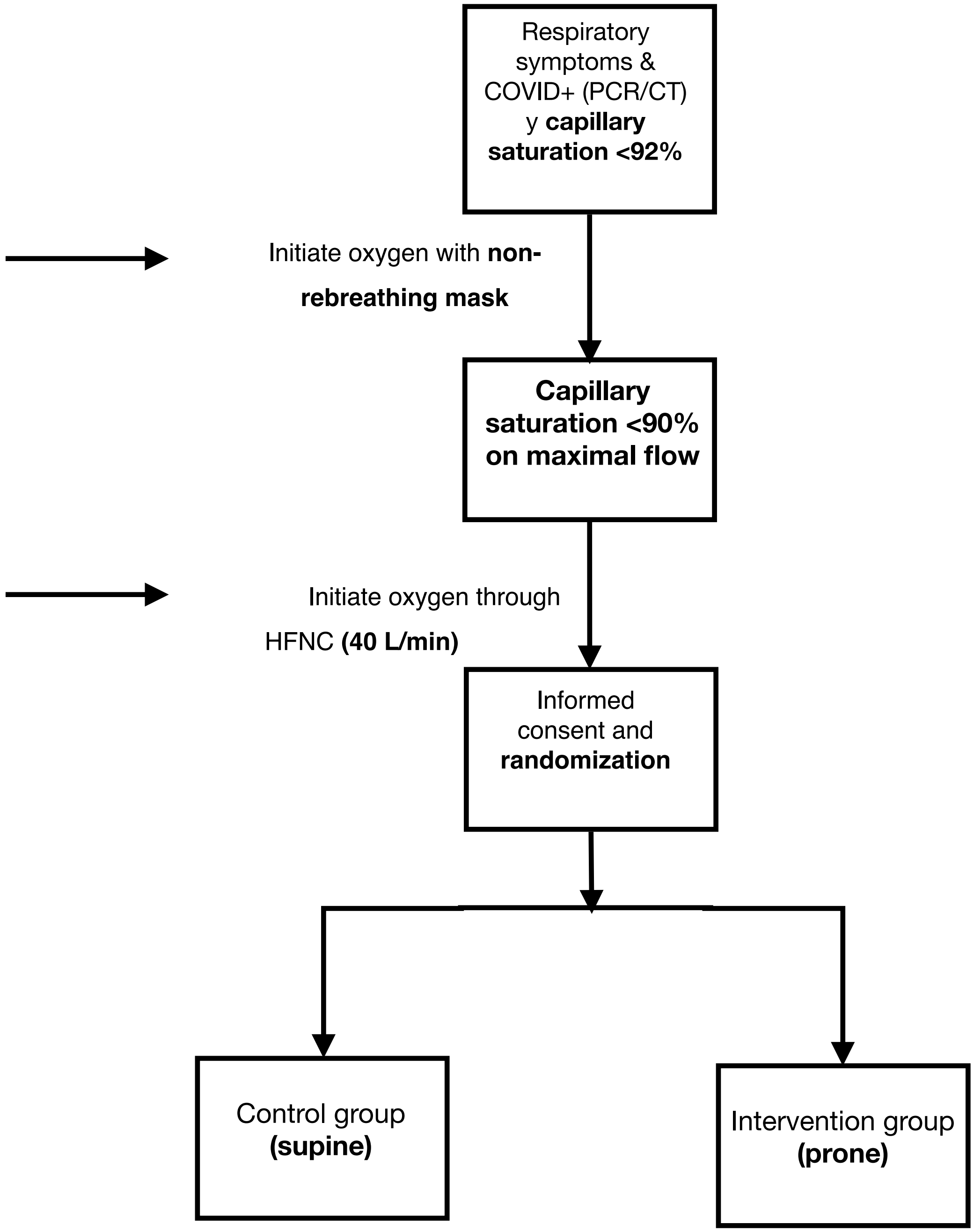

Endotracheal intubation for all patients whose **capillary saturation is ≤80% despite 100% inspired oxygen** and maximal tolerated flow, as well as patients with CO2 retention, **progressive clinical worsening in respiratory distress**, anxiety or altered mental status, regardless the magnitude of oxygen support.

Consentimiento Informado

Nombre del Investigador Principal: Dr. Miguel Ángel Ibarra Estrada

Este Formulario de Consentimiento Informado se dirige a hombres y mujeres que son atendidos en los hospitales mencionados en el encabezado, y que se les invita a participar en la investigación clínica Uso de posición prona en pacientes despiertos con síndrome de dificultad respiratoria aguda moderado/severo y COVID-19

Introducción

Yo soy el Dr. Miguel Ángel Ibarra Estrada, trabajo para el Instituto Jalisciense de Cancerología, el Hospital Civil de Guadalajara, y el Consejo Nacional de Ciencia y Tecnología. Estamos investigando sobre el uso de un método no ventilatorio considerado actualmente como "no convencional" en pacientes con Síndrome de Falla Respiratoria Aguda asociada a COVID-19, una entidad muy común a nivel global actualmente. Le voy a dar información e invitarle a participar de esta investigación. No tiene que decidir hoy si participar o no en esta investigación. Antes de decidirse, puede hablar con alguien con quien se sienta cómodo sobre la investigación.

Puede que haya algunas palabras que no entienda. Por favor, interrúmpame según le informo para darme tiempo a explicarle. Si tiene preguntas más tarde, puede preguntarme a mi, a otros doctores que investigan o a miembros del equipo médico.

Propósito

El Síndrome de Dificultad Respiratoria Aguda asociada a COVID-19 es un estado de gravedad asociado a una alteración severa en los pulmones, que no permite que el oxígeno que se respira se traslade a la sangre. A pesar de las mejoras en el tratamiento especializado en las últimas décadas, aproximadamente 6-8 de cada 10 de los pacientes que son intubados, fallecen. Existe un modo no ventilatorio ya conocido, aunque poco utilizado en nuestro país, que podría favorecer el mejoramiento de este estado de gravedad y potencialmente evitar la intubación y que usted o su familiar tengan que recibir soporte ventilatorio mecánico. El determinar con certeza el beneficio potencial de este método es la razón por la que hacemos este estudio.

Tipo de Intervención de Investigación

Esta investigación incluirá el manejo no ventilatorio llamado "pronación", que consiste en voltearlo a usted o a su familiar 'boca abajo' la mayor parte del día según lo tolere, cuando el equipo médico identifique que se encuentra en riesgo alto de requerir ventilación mecánica, y se llevará a cabo desde la identificación de la alteración pulmonar moderada/severa hasta la resolución o desenlace final.

Selección de participantes

Estamos invitando a todos los adultos con Síndrome de Dificultad Respiratoria Aguda asociada a COVID-19 para participar en la investigación sobre un método no ventilatorio alternativo con potencial para mejorar su pronóstico.

Participación Voluntaria

Su participación (o la de su familiar) en esta investigación es totalmente voluntaria. Usted puede elegir participar o no hacerlo. Tanto si elige participar o no, continuarán todos los servicios que reciba en esta unidad y nada cambiará. Usted puede cambiar de idea más tarde y dejar de participar aún cuando haya aceptado antes.

Información sobre el método de investigación

El método de pronación es un método aplicado desde hace más de 20 años a los pacientes con síndrome de dificultad respiratoria aguda. Se ha probado antes con personas con este tipo de enfermedad pulmonar, aunque solo en los que ya estuvieron recibiendo ventilación mecánica, demostrando resultados favorables al grado de volverse una recomendación ampliamente aceptada en todo el mundo. Ahora queremos probar este método en personas de nuestro entorno con insuficiencia

respiratoria asociada a COVID-19 pero desde antes de ser intubados. A esta investigación se la denomina “Fase 2” de un ensayo clínico.

Debe saber que aunque cualquier tipo de manejo de pacientes graves tiene efectos adversos potenciales, de este método en particular solo se han reportado eventualidades no serias como ulceraciones o edema en cara, o dolor transitorio en brazos o espalda.

Algunos de los participantes en la investigación no serán tratados con dicho método. En su lugar, recibirán el manejo estándar que es básicamente el recomendados actualmente según autoridades internacionales.

Procedimientos y Protocolo

Necesitamos verificar la eficacia y seguridad de la terapia de pronación. Para hacer esto, pondremos a los participantes en dos grupos. Los grupos son seleccionados por azar, al igual como lanzar una moneda al aire.

Los trabajadores de la salud le estarán observando cuidadosamente y también a los otros participantes durante el estudio. Si llega a preocuparnos lo que la pronación hace, podremos realizar cambios inmediatos en el manejo. Si existe algo que le preocupe o que le moleste sobre la investigación, por favor hable conmigo o con alguno de los otros investigadores. Usted recibirá el resto del tratamiento de su condición bajo pautas internacionales.

Duración

El seguimiento que se le dará a usted o a su familiar incluirá toda su estadía en la unidad COVID del Hospital Civil Fray Antonio Alcalde y hasta su egreso.

Efectos Secundarios

Como ya se mencionó, este método ventilatorio no tiene efectos adversos serios. Es posible que pueda también causar problemas que aún no conocemos. Sin embargo, le haremos un seguimiento y mantendremos un registro de cualquier efecto no deseado o cualquier problema. Puede que usemos otros métodos para disminuir los síntomas de los efectos secundarios o reacciones. O puede que dejemos de usar el método experimental. Si esto es necesario, lo discutiremos con usted y siempre se le consultará antes de continuar con el próximo paso.

Beneficios

Si usted o su familiar participa en esta investigación, no se garantiza que obtenga ningún beneficio personal. Puede que no halla beneficio para usted, pero es probable que su participación nos ayude a encontrar una respuesta a la pregunta de investigación. Puede que no haya beneficio para la sociedad en la fase actual de la investigación, pero es probable que generaciones futuras se beneficien.

Confidencialidad

Con esta investigación, se realiza algo fuera de lo ordinario en su comunidad. Es posible que si otros miembros de la comunidad saben que usted participa, puede que le hagan preguntas. Nosotros no compartiremos la identidad de aquellos que participen en la investigación. La información que recojamos por este proyecto de investigación se mantendrá confidencial. La información acerca de usted o su familiar que se recogerá durante la investigación será puesta fuera de alcance y nadie además de los investigadores tendrán acceso a ella. Cualquier información acerca de usted tendrá un número en lugar de su nombre. Solo los investigadores sabrán cual es su número y se mantendrá la información encerrada en cabina con llave. No será compartida ni entregada a nadie.

Derecho a negarse o retirarse

Usted no tiene porque participar en esta investigación si no desea hacerlo, y el negarse a participar no le afectará en ninguna forma a que sea tratado en esta unidad. Usted aún tendrá todos los beneficios que de otra forma tendría. Puede dejar de participar en la investigación en cualquier momento que desee sin perder sus derechos como paciente aquí. Su tratamiento no será afectado en ninguna forma.

A Quién Contactar

Si tiene cualquier pregunta puede hacerlas ahora o más tarde, incluso después de haberse iniciado el estudio. Si desea hacer preguntas más tarde, puede contactar cualquiera de las siguientes personas: Miguel Ángel Ibarra Estrada, Guadalupe Aguirre Avalos, Quetzalcóatl Chávez Peña (Hospital Civil Fray Antonio Alcalde, Unidad de Terapia Intensiva).

Formulario de Consentimiento

He sido invitado a participar en la investigación de un método no ventilatorio relativamente nuevo llamado pronación, para el manejo del Síndrome de Dificultad Respiratoria Aguda asociada a COVID-19. He sido informado de que los riesgos asociados directamente al manejo son mínimos. Se me ha proporcionado el nombre de uno o más investigadores que pueden ser contactados en caso de requerir mayor información.

He leído la información proporcionada o me ha sido leída. He tenido la oportunidad de preguntar sobre ella y se me han contestado satisfactoriamente las preguntas que he realizado. Consiento voluntariamente participar (o que mi familiar participe) en esta investigación, y entiendo que tengo el derecho de retirarme de la investigación en cualquier momento sin que afecte en ninguna manera mi cuidado médico (o de mi familiar).

Nombre del Participante ____

Nombre y firma del participante o representante legal ____________________________________

Fecha (día/mes/año) / /

Iniciales del investigador o asistente:_______________

Formato escrito para registro de datos

Protocolo de estudio: **Pronación en pacientes no intubados con falla respiratoria asociada a COVID-19**

Datos generales							
**Iniciales**				**Edad**		**Género**	
**Registro hospitalario**				**Grupo (control o intervención)**		**Altura (cm)**	
**Peso (kg)**		**Diabetes tipo 2**		**Hipertensión**		**Enf. coronaria**	
**Cáncer**		**Enf. Autoimune**		**Enf. Pulmonar crónica**		**Enf. renal crónica**	
**Enf. Vasc. Periférica**		**Enf. Vasc. Cerebral**		**Uso de IECAS**		**Uso de ARAII**	
**COVID confirmado**		**Uso previo de esteroides**		**Días de síntomas antes de primera pronación**		**Llegó como "hipoxemia feliz" (sin disnea)**	
**Terapia Intensiva o Intermedia**		**APACHE II**					
**Mediciones basales (aleatorización)**							
**Sat O2 inicial (%)**		**FiO2 inicial**		**pCO2 inicial**		**FR inicial**	
**FC inicial**		**PAM inicial (mmHg)**		**pO2/FiO2 inicial**		**Flujo de CNAF inicial**	
**Índice ROX inicial**		**Sa/FiO2 inicial**		**LUS score inicial**			
**Tratamiento Farmacológico**							
**Antivirales**		**Esteroide**		**Anticoagulación terapéutica**		**Bloqueador de IL-6**	

**Escalada de terapia**							
**Tiempo a VNI (si se utilizó)**		**Máxima FiO2 (en CNAF o VNI)**		**Máximo flujo (en CNAF)**		**Máxima PEEP (en VNI)**	
**FR previa a intubación**		**Saturación previa a intubación**		**FiO2 previo a intubación**		**Índice ROX previo a intubación**	
**Efectos adversos**							
**Empeoramiento de disnea**		**Retiro accidental de líneas IV**		**Retiro accidental de sondas**		**Retiro accidental de CVC**	
**Ulceraciones**		**Dolor/ incomodidad**		**Vómito**		**Trombosis venosa profunda**	
**Desenlaces**							
**Días totales de prono**		**Días totales de CNAF**		**Días totales de VMNI**		**Intubación**	
**Causa de la intubación** (aumento en disnea, desaturación o alt. edo. alerta)		**Tiempo a intubación (días)**		**Traqueostomía**		**Muerte en UTI (hasta 60 días)**	
**Tiempo a muerte (días)**		**Días libres de ventilación mecánica (28 días)**		**Días de estancia en UTI**		**Días de estancia hospitalaria**	
**Muerte hospitalaria (hasta 60 días)**		**Violación al protocolo (cruce de grupos, anotar la razón)**					

Pronación																												
**pO2/FiO2 después de primera sesión de prono (1 h)**		**FR después de primera sesión de prono (1 h)**		**Sa/FiO2 después de primera sesión de prono (1 h)**		**Índice ROX después de primera sesión de prono (1 h)**																						
**Días**	**1**	**2**	**3**	**4**	**5**	**6**	**7**	**8**	**9**	**10**	**11**	**12**	**13**	**14**	**15**	**16**	**17**	**18**	**19**	**20**	**21**	**22**	**23**	**24**	**25**	**26**	**27**	**28**
**Horas totales de prono**																												
**# de sesiones**																												
**Tiempo de la sesión más prolongada**																												
**Laboratoriales**																												
**Hb**																												
**Leu**																												
**Plaq**																												
**Na**																												
**K**																												
**Bilirrubinas**																												
**Creatinina**																												
**Dímero D**																												
**Troponinas I**																												
**Lactato**																												
**Procalcitonina**																												
