# Supplementary material for: Prone positioning in non-intubated patients with COVID-19 associated acute respiratory failure, the PRO-CARF trial: A structured summary of a study protocol for a randomised controlled trial
Source: Trials. 2020 Nov 23;21:940. doi: 10.1186/s13063-020-04882-2 (PMC7680981; doi:10.1186/s13063-020-04882-2)
Supplement: Supplementary file 2 — Additional file 2 [file 13063_2020_4882_MOESM2_ESM.doc]

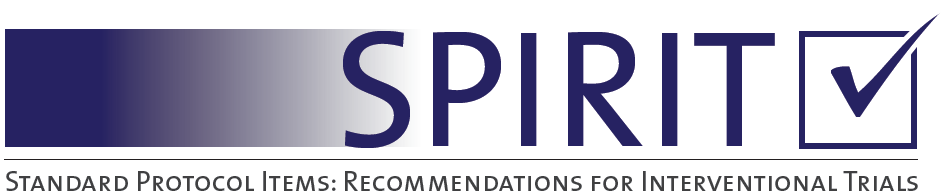


SPIRIT 2013 Checklist: Recommended items to address in a clinical trial protocol and related documents*

| Section/item | ItemNo | Description |
| --- | --- | --- |
| **Administrative information** | | |
| Title | 1 | Prone Positioning in Non-intubated Patients With COVID-19 Associated Acute Respiratory Failure (PRO-CARF). *please see page 1* |
| Trial registration | 2a | *Study protocol registered at* [*www.clinicaltrials.gov*](http://www.clinicaltrials.gov/) *with # NCT* 04477655, *please see page 7* |
|  | 2b | All items from the World Health Organization Trial Registration Data Set. *We have followed all the items of* [*www.clinicaltrials.gov*](http://www.clinicaltrials.gov/)*. Please see page 7* |
| Protocol version | 3 | *Research Protocol version 2.0 dated May 3rd, 2020, please see page 1* |
| Funding | 4 | Not applicable |
| Roles and responsibilities | 5a | Names, affiliations, and roles of protocol contributors, please see page 1 for details |
|  | 5b | Name and contact information for the trial sponsor. Not applicable |
|  | 5c | Role of study sponsor and funders, if any. Not applicable |
|  | 5d | Composition, roles, and responsibilities of the coordinating centre, steering committee, endpoint adjudication committee, data management team, and other individuals or groups overseeing the trial, if applicable. Please see page 5 |
| Introduction |  |  |
| Background and rationale | 6a | Description of research question and justification for undertaking the trial, including summary of relevant studies (published and unpublished) examining benefits and harms for each intervention. Please see page 2 for details. |
|  | 6b | Explanation for choice of comparators. Please see page 3 for details. |
| Objectives | 7 | Specific objectives or hypotheses. Please see page 3. |
| Trial design | 8 | Description of trial design including type of trial (eg, parallel group, crossover, factorial, single group), allocation ratio, and framework (e.g. superiority, equivalence, non-inferiority, exploratory). Please see pages 4 and 5 for details. |
| Methods: Participants, interventions, and outcomes | | |
| Study setting | 9 | Description of study settings (eg, community clinic, academic hospital) and list of countries where data will be collected. Reference to where list of study sites can be obtained. Please see page 4 for details. |
| Eligibility criteria | 10 | Inclusion and exclusion criteria for participants. If applicable, eligibility criteria for study centres and individuals who will perform the interventions (eg, surgeons, psychotherapists). Please see page 4 for details. |
| Interventions | 11a | Interventions for each group with sufficient detail to allow replication, including how and when they will be administered. Please see page 5 for details |
|  | 11b | Criteria for discontinuing or modifying allocated interventions for a given trial participant (eg, drug dose change in response to harms, participant request, or improving/worsening disease). Please see page 5 for details. |
|  | 11c | Strategies to improve adherence to intervention protocols, and any procedures for monitoring adherence (eg, drug tablet return, laboratory tests). Please see page 5 for details. |
|  | 11d | Relevant concomitant care and interventions that are permitted or prohibited during the trial. Please see page 5 for details. |
| Outcomes | 12 | Primary, secondary, and other outcomes, including the specific measurement variable (eg, systolic blood pressure), analysis metric (eg, change from baseline, final value, time to event), method of aggregation (eg, median, proportion), and time point for each outcome. Explanation of the clinical relevance of chosen efficacy and harm outcomes is strongly recommended. Please see page 3 for details. |
| Participant timeline | 13 | Time schedule of enrolment, interventions (including any run-ins and washouts), assessments, and visits for participants. A schematic diagram is highly recommended. Please see page 6 for details. |
| Sample size | 14 | Estimated number of participants needed to achieve study objectives and how it was determined, including clinical and statistical assumptions supporting any sample size calculations. Please see page 7 for details. |
| Recruitment | 15 | Strategies for achieving adequate participant enrolment to reach target sample size. Not applicable. |
| **Methods: Assignment of interventions (for controlled trials)** | | |
| Allocation: |  |  |
| Sequence generation | 16a | Method of generating the allocation sequence (eg, computer-generated random numbers), and list of any factors for stratification. To reduce predictability of a random sequence, details of any planned restriction (eg, blocking) should be provided in a separate document that is unavailable to those who enrol participants or assign interventions. Please see page 6 for details. |
| Allocation concealment mechanism | 16b | Mechanism of implementing the allocation sequence (eg, central telephone; sequentially numbered, opaque, sealed envelopes), describing any steps to conceal the sequence until interventions are assigned. Please see page 6 for details. |
| Implementation | 16c | Who will generate the allocation sequence, who will enrol participants, and who will assign participants to interventions. Please see page 6 for details. |
| Blinding (masking) | 17a | Who will be blinded after assignment to interventions (eg, trial participants, care providers, outcome assessors, data analysts), and how. Please see page 6 for details. |
|  | 17b | If blinded, circumstances under which un-blinding is permissible, and procedure for revealing a participant’s allocated intervention during the trial. Not applicable. |
| **Methods: Data collection, management, and analysis** | | |
| Data collection methods | 18a | Plans for assessment and collection of outcome, baseline, and other trial data, including any related processes to promote data quality (eg, duplicate measurements, training of assessors) and a description of study instruments (eg, questionnaires, laboratory tests) along with their reliability and validity, if known. Reference to where data collection forms can be found, if not in the protocol. Please see page 6 and 14 for details. |
|  | 18b | Plans to promote participant retention and complete follow-up, including list of any outcome data to be collected for participants who discontinue or deviate from intervention protocols. Please see page 6 for details. |
| Data management | 19 | Plans for data entry, coding, security, and storage, including any related processes to promote data quality (eg, double data entry; range checks for data values). Reference to where details of data management procedures can be found, if not in the protocol. Please see page 6 for details. |
| Statistical methods | 20a | Statistical methods for analysing primary and secondary outcomes. Reference to where other details of the statistical analysis plan can be found, if not in the protocol. Please see page 7 for details. |
|  | 20b | Methods for any additional analyses (eg, subgroup and adjusted analyses). Not applicable. |
|  | 20c | Definition of analysis population relating to protocol non-adherence (eg, as randomised analysis), and any statistical methods to handle missing data (eg, multiple imputation). Not applicable. |
| **Methods: Monitoring** | | |
| Data monitoring | 21a | Composition of data monitoring committee (DMC); summary of its role and reporting structure; statement of whether it is independent from the sponsor and competing interests; and reference to where further details about its charter can be found, if not in the protocol. Alternatively, an explanation of why a DMC is not needed………… Please see page 6 for details. |
|  | 21b | Description of any interim analyses and stopping guidelines, including who will have access to these interim results and make the final decision to terminate the trial. Please see page 7 for details. |
| Harms | 22 | Plans for collecting, assessing, reporting, and managing solicited and spontaneously reported adverse events and other unintended effects of trial interventions or trial conduct. Please see page 7 for details. |
| Auditing | 23 | Frequency and procedures for auditing trial conduct, if any, and whether the process will be independent from investigators and the sponsor. Not applicable. |
| Ethics and dissemination | | |
| Research ethics approval | 24 | Plans for seeking research ethics committee/institutional review board (REC/IRB) approval. Please see page 7 for details. |
| Protocol amendments | 25 | Plans for communicating important protocol modifications (eg, changes to eligibility criteria, outcomes, analyses) to relevant parties (eg, investigators, REC/IRBs, trial participants, trial registries, journals, regulators). Please see page 7 for details. |
| Consent or assent | 26a | Who will obtain informed consent or assent from potential trial participants or authorised surrogates, and how (see Item 32). Please see page 6 for details. |
|  | 26b | Additional consent provisions for collection and use of participant data and biological specimens in ancillary studies, if applicable. Not applicable. |
| Confidentiality | 27 | How personal information about potential and enrolled participants will be collected, shared, and maintained in order to protect confidentiality before, during, and after the trial. Please see page 6 for details. |
| Declaration of interests | 28 | Financial and other competing interests for principal investigators for the overall trial and each study site. Please see page 8 for details. |
| Access to data | 29 | Statement of who will have access to the final trial dataset, and disclosure of contractual agreements that limit such access for investigators. Please see page 8 for details. |
| Ancillary and post-trial care | 30 | Provisions, if any, for ancillary and post-trial care, and for compensation to those who suffer harm from trial participation. Not applicable. |
| Dissemination policy | 31a | Plans for investigators and sponsor to communicate trial results to participants, healthcare professionals, the public, and other relevant groups (eg, via publication, reporting in results databases, or other data sharing arrangements), including any publication restrictions…. Please see page 8 for details. |
|  | 31b | Authorship eligibility guidelines and any intended use of professional writers. Not applicable. |
|  | 31c | Plans, if any, for granting public access to the full protocol, participant-level dataset, and statistical code. Please see page 8 for details. |
| Appendices |  |  |
| Informed consent materials | 32 | Model consent form and other related documentation given to participants and authorised surrogates…...please see page 11. |
| Biological specimens  Miscellaneous | 33  34 | Plans for collection, laboratory evaluation, and storage of biological specimens for genetic or molecular analysis in the current trial and for future use in ancillary studies, if applicable. Not applicable.  Any other relevant information. Not applicable. |

*It is strongly recommended that this checklist be read in conjunction with the SPIRIT 2013 Explanation & Elaboration for important clarification on the items. Amendments to the protocol should be tracked and dated. The SPIRIT checklist is copyrighted by the SPIRIT Group under the Creative Commons “[Attribution-NonCommercial-NoDerivs 3.0 Unported](http://www.creativecommons.org/licenses/by-nc-nd/3.0/)” license.
